# HYDAMTIQ, a selective PARP‐1 inhibitor, improves bleomycin‐induced lung fibrosis by dampening the TGF‐β/SMAD signalling pathway

**DOI:** 10.1111/jcmm.12967

**Published:** 2016-10-04

**Authors:** Laura Lucarini, Mariaconcetta Durante, Cecilia Lanzi, Alessandro Pini, Giulia Boccalini, Laura Calosi, Flavio Moroni, Emanuela Masini, Guido Mannaioni

**Affiliations:** ^1^ Department of Neuroscience, Psychiatry, Drug Area and Child Health (NEUROFARBA)Section of Pharmacology and ToxicologyUniversity of FlorenceFlorenceItaly; ^2^ Department of Experimental and Clinical MedicineSection of Anatomy and HistologyUniversity of FlorenceFlorenceItaly

**Keywords:** HYDAMTIQ, PAO, ROS, αSMA, SMAD

## Abstract

Idiopathic pulmonary fibrosis is a severe disease characterized by excessive myofibroblast proliferation, extracellular matrix and fibrils deposition, remodelling of lung parenchyma and pulmonary insufficiency. Drugs able to reduce disease progression are available, but therapeutic results are unsatisfactory; new and safe treatments are urgently needed. Poly(ADP‐ribose) polymerases‐1 (PARP‐1) is an abundant nuclear enzyme involved in key biological processes: DNA repair, gene expression control, and cell survival or death. In liver and heart, PARP‐1 activity facilitates oxidative damage, collagen deposition and fibrosis development. In this study, we investigated the effects of HYDAMTIQ, a potent PARP‐1 inhibitor, in a murine model of lung fibrosis. We evaluated the role of PARP on transforming growth factor‐β (TGF‐β) expression and TGF‐β/SMAD signalling pathway in lungs. Mice were intratracheally injected with bleomycin and then treated with either vehicle or different doses of HYDAMTIQ for 21 days. Airway resistance to inflation and lung static compliance, markers of lung stiffness, were assayed. Histochemical and biochemical parameters to evaluate TGF‐β/SMAD signalling pathway with alpha‐smooth muscle actin (αSMA) deposition and the levels of a number of inflammatory markers (tumour necrosis factor‐α, interleukin‐1β, iNOS and COX‐2) were performed. Bleomycin administration increased lung stiffness. It also increased lung PARP activity, TGF‐β levels, pSMAD3 expression, αSMA deposition and content of inflammatory markers. HYDAMTIQ attenuated all the above‐mentioned physiological, biochemical and histopathological markers. Our findings support the proposal that PARP inhibitors could have a therapeutic potential in reducing the progression of signs and symptoms of the disease by decreasing TGF‐β expression and the TGF‐β/SMAD transduction pathway.

## Introduction

Idiopathic pulmonary interstitial fibrosis (IPF) is the most common fibrotic disease of the lungs with a poor prognosis and a median survival of ~3 years after the diagnosis 1. While its aetiology is still unknown, it has been proposed that the disease is due to an increased proliferation of fibroblasts with excessive formation and accumulation of collagen and other extracellular matrix components, such as alpha‐smooth muscle actin (αSMA) and fibronectin, with abnormal remodelling of damaged lungs. This accumulation is associated with alveolar injury, airway stiffening, thickening of the air–blood membrane, chronic inflammation and eventually progressive respiratory failure 2, 3. Until a few years ago, corticosteroids in combination with immune‐suppressants and free radical scavengers have been the mainstay of therapy for IPF, but a recent clinical trial aimed at evaluating the effects of a treatment with prednisone plus azathioprine, and N‐acetylcysteine showed an increased incidence of death and hospitalization in treated patients 4. Indeed, the 2015 Official ATS/ERS/JRS/ALAT Clinical Practice Guideline 5 provided strong recommendation against using this harmful combination.

The observation that it is possible to delay disease progression using either pirfenidone, a compound able to reduce the expression of transforming growth factor‐β (TGF‐β), or nintedanib, an inhibitor of several tyrosine kinases, including platelet‐derived growth factor receptor, fibroblast growth factor and VEGF receptors, brought new hope for a successful medical treatment of the disease 6, 7. Both pirfenidone and nintedanib have been recently approved for IPF treatment 8, 9. However, the therapeutic effects of these agents are not completely satisfactory, and the identification of original targets followed by possible development of new drugs is urgently needed 10, 11. Along this line, it is interesting to mention that an excessive expression of TGF‐β is present in the lungs of IPF patients and that an excessive signalling through the TGF‐β/SMAD pathway, which leads to an increased formation of collagen and other extracellular matrix components in fibroblast or smooth muscle cells, seems thus contributing to the pathogenesis of lung fibrosis 12, 13, 14. Moreover, TGF‐β/SMAD3 signalling pathway and the dependent expression of SMAD target genes require poly(ADP‐ribose) polymerases‐1 (PARP‐1) activation 15, 16.

PARP‐1 is the most abundant and studied member of a group of enzymes leading to poly(ADP‐ribosyl)ation (PARylation), a post‐translational modification of protein 17, 18. It is mostly located in cell nuclei and is strongly activated by DNA strand breaks due to reactive oxygen species 19. PARP‐1 uses NAD^+^ as the ADP donor to generate long poly(ADP‐ribose) polymers covalently attached to suitable acceptor proteins, such as histones, transcription factors and PARP‐1 itself. PARPs plays key roles in the maintenance of genomic integrity, epigenetic regulation of gene expression, control of cell cycle and cell death 20, 21, 22.

Gene targeting approaches and the use of non‐selective inhibitors have shown that PARP‐1 is involved in a number of fibrotic disorders affecting heart 23, 24, liver 25, vessels 15, 26 and lungs 27, 28. Particularly interesting is the observation that PARylated protein levels are significantly increased in lung fibroblasts isolated from IPF patients 16. Moreover, PARP inhibitors not only prevented the development of fibrosis but also reduced collagen accumulation and restored liver function in an accepted model of liver fibrosis 25. However, the molecular mechanisms of the effects of PARP inhibitor in fibrosis are not completely elucidated, and in this study, we hypothesized that PARP‐1 activation, by facilitating the TGF‐β/SMAD3 transduction pathway, is involved in the process of fibroblast activation, αSMA and collagen accumulation and finally fibrosis. We tested this hypothesis in bleomycin‐induced lung pathology in mice, a commonly used and well‐characterized animal model to evaluate the possible efficacy of drugs in inflammatory and fibrotic lung diseases 29, 30. In order to reduce lung PARP‐1 activity, we used 2‐dimethylaminomethyl‐9‐hydroxy‐thieno[2,3‐c]isoquinolin‐5(4H)‐one) (HYDAMTIQ) a potent PARP‐1/2 inhibitor (IC_50_ 2–20 nM), previously characterized in models of brain ischaemia–reperfusion damage and of ovalbumin‐induced asthma in guinea pigs 31, 32, 33.

## Materials and methods

### Drugs and reagents

HYDAMTIQ, 2‐((dimethylamino)methyl)‐9‐hydroxythieno[2,3‐c]isoquinolin‐5(4H)‐one (M.W. 310.8), was synthesized as previously reported 34 and kindly provided by Roberto Pellicciari, from the University of Perugia, Italy. This compound was tested against a panel of 62 receptors and enzymes at a concentration of 10 μM (NOVASCREEN) and showed an excellent selectivity towards PARP‐1 and PARP‐2 34.

The compound was >97% pure as assessed by HPLC. HYDAMTIQ (as hydrochloride salt) was dissolved in 1× PBS plus 10% DMSO and 5% ethanol. Bleomycin (Merck‐Millipore, Milan, Italy) (0.05 IU) was dissolved in 50 μl of saline.

### Animals

Male C57BL/6 mice, 2 months old and weighing 25–30 g, were used for the experiments. They were purchased from a commercial dealer (Harlan, Udine, Italy), fed with standard diet and water ad libitum, and housed in a controlled environment at 22°C with a 12‐hr light/dark cycle. Animal studies were conducted at Centre for Laboratory Animal Housing and Experimentation (CeSAL) of the University of Florence. The experimental protocols were designed in compliance with the Italian and the European Community regulations on animal experimentation for scientific purposes (D.M. 116192; O.J. of E.C. L358/1 12/18/1986). The experimental protocols were approved by the Ethical Committee of the University of Florence, Italy.

### Surgery and treatments

Eighty‐eight mice were anaesthetized with zolazepam/tiletamine (Zoletil, 50/50 mg/ml; Virbac Srl, Milan, Italy; 50 μg/g i.p. in 100 μl of saline); 72 of them were treated with bleomycin (0.05 IU in 50 μl of saline), and the other 16 were treated with 50 μl of saline (referred to as non‐fibrotic negative controls, Naïve), both delivered by intratracheal injection.

The 72 animals, previously treated with bleomycin, were randomly divided into four groups. Three groups were treated with two daily intra‐peritoneal injection of 100 μl HYDAMTIQ solution (1, 3 and 10 mg/kg/day), immediately after bleomycin administration and for the next 21 days, before undergoing the pressure at airway opening (PAO) test. These are referred to as bleomycin+HYDAMTIQ‐treated groups. The fourth group was treated only with vehicle and referred as fibrotic positive controls (bleomycin+vehicle).

### Functional assay of fibrosis

At day 21 after surgery, the mice were subjected to measurement of airway resistance to inflation and static lung compliance, functional parameters related to fibrosis‐induced lung stiffness, using a constant volume mechanical ventilation method with constant number of breaths in a minute, and in case of static compliance determination, a positive end‐expiratory pressure of 3 cm H_2_O was applied, to mimic spontaneous ventilation 35, 36, 37. Briefly, upon anaesthesia (zolazepam/tiletamine, 50 μg/g i.p. in 100 μl of saline), the mice were operated to insert a 22‐gauge cannula (0,8 mm diameter, Venflon 2; Viggo Spectramed, Windlesham, UK) into the trachea and then ventilated with a small‐animal respirator (Ugo Basile, Comerio, Italy), adjusted to deliver a tidal volume of 0.8 ml at a rate of 20 strokes/min. Changes in lung resistance to inflation (PAO) were registered by a high‐sensitivity pressure transducer (P75 type 379; Hugo Sachs Elektronik, March‐Hugstetten, Germany) connected to a polygraph (Harvard Apparatus, Holliston, MA, USA) at the following settings: gain 1, chart speed 25 mm/sec. Inflation pressure was measured for at least 3 min. In each mouse, PAO measurements (expressed as mm on the chart) were carried out on at least 40 consecutive tracings of respiratory strokes and then averaged. For static lung compliance determination, multiple linear regression was used to fit measured pressure and volume in each individual mouse to the linear model of the lung 37.

### Lung tissue sampling

After the functional assay, the animals were killed with lethal dose of anaesthetic drugs, and the whole left lungs were excised and fixed by immersion in 4% formaldehyde in phosphate‐buffered saline for histological analysis. The right lungs were weighed, quickly frozen and stored at −80°C. At the moment of the biochemical measurements, the samples were thawed at 4°C, homogenized on ice in 50 mM Tris‐HCl buffer containing 180 mM KCl and 10 mM ethylenediaminetetracetic acid (EDTA), pH 7.4, and then centrifuged at 10,000 × g, 4°C, for 30 min., unless otherwise reported. The supernatants and the pellets were collected and used for separate assays as detailed below.

### Hydroxyproline analysis

The frozen tissue were previously lyophilized for 48 hrs and then thoroughly homogenized in distilled water. The samples were gently mixed with 12 M hydrochloric acid and hydrolysed by autoclaving at 120°C for 40 min. in O'‐ring screw‐capped Nalgene high‐temperature polypropylene tubes of 2‐ml capacity. Chloramine T reagent was added to the hydrolyzate, and the oxidation was allowed to proceed for 25 min. at room temperature. Finally, Ehrlich's aldehyde reagent was added to the samples and incubated at 65°C for 20 min., and the absorbance was read at 550 nm.

### Western blot analysis for PARylated protein content

Lung tissues were homogenized in 500 μl of radio immunoprecipitation assay buffer plus protease inhibitors and centrifuged at 12,000 × g for 5 min. The supernatants were transferred in tube, and total protein levels were quantified using Protein Assay of Pierce (Rockford, IL, USA). Thirty micrograms of proteins was subjected to Western blot analysis, using a mouse monoclonal anti‐PAR(10H) antibody (Alexis Biochemicals, Florence, Italy) diluted 1:1000 in PBS‐T containing either 5% non‐fat dry milk. This monoclonal antibody recognizes poly(ADP‐ribose) synthesized by PARP enzymes. The binding of the primary antibody was determined by the addition of suitable peroxidase‐conjugated secondary antibodies (anti‐mouse antibody 1:5000). Densitometric analyses were done with the Quantity One analysis software (Bio‐Rad, Hercules, CA, USA).

### Histology assessment of collagen deposition, goblet cell hyperplasia and smooth muscle layer thickness

Histological sections, 6 μm thick, were cut from paraffin‐embedded lung samples and stained with haematoxylin and eosin for routine observations and periodic acid‐Schiff (PAS) or modified Azan method 38 for the evaluation of goblet cells and collagen deposition. Staining was performed in a single session to minimize the artifactual differences in collagen staining. For each mouse, 20 photomicrographs of peribronchial connective tissue were randomly taken, applying a systematic uniform random sampling method 39, and using a digital camera connected to a light microscope with a 40× objective. Measurements of optical density (OD) of aniline blue‐stained collagen fibres were carried out using ImageJ 1.33 image analysis program (http://rsb.info.nih.gov/ij), upon appropriate threshold to exclude aerial air spaces and bronchial/alveolar epithelium, as previously described 40. Values are mean ± S.E.M. of the OD measurements (arbitrary units) of individual mouse from the different experimental groups.

For morphometry of smooth muscle layer thickness and bronchial goblet cell numbers, both key markers of airway remodelling and lung tissue sections were stained with haematoxylin and eosin and with PAS, respectively. Digital photomicrographs of medium‐ and small‐sized bronchi were randomly taken 39. Measurements of the thickness of the bronchial smooth muscle layer were carried out on the digitized images using the above‐mentioned software. Periodic acid‐Schiff‐stained goblet cells and total bronchial epithelial cells were counted on bronchial cross‐section profiles, and the percentage of goblet cells was calculated. For both parameters, values are mean ± S.E.M. of individual mice (at least 20 images each) from the different experimental groups.

### Determination of αSMA deposition in immunofluorescence

Immunofluorescence analysis was performed as previously described 41. Briefly, lung sections were deparaffinized, boiled for 10 min. in sodium citrate buffer (10 mM, pH 6.0; Bio‐Optica, Milan, Italy) for antigen retrieval and immunostained with rabbit monoclonal anti‐αSMA antibody (1:200; Abcam, Cambridge, UK) followed by goat anti‐rabbit Alexa Fluor 568‐conjugated IgG (1:300; Invitrogen, San Diego, CA, USA). Negative controls were performed with non‐immune rabbit serum substituted for the primary antibodies. After counterstaining with 4′,6‐diamidino‐2‐phenylindole (DAPI), representative images were acquired by an Olympus BX63 microscope coupled to CellSens Dimension Imaging Software version 1.6 (Olympus, Milan, Italy). To quantify αSMA expression, densitometric analysis of the intensity of fluorescence signal was performed on digitized images using ImageJ software (http://rsbweb.nih.gov/ij). Twenty regions of interest were evaluated for each sample. Values are expressed as mean ± S.E.M. of the OD measurements (arbitrary units) of individual mouse from the different experimental groups.

### Determination of TGF‐β, interleukin 1β and tumour necrosis factor‐α

The levels of TGF‐β, the major profibrotic cytokine involved in fibroblast activation, and two pro‐inflammatory cytokines, interleukin (IL)‐1β and tumour necrosis factor (TNF)‐α, were measured on aliquots (20 μl) of lung homogenate supernatants by using the FlowCytomix assay (Bender Medsystems GmbH, Vienna, Austria), following the protocol provided by the manufacturer. In brief, suspensions of anti‐TGF‐β, IL‐1 β or TNF‐α‐coated beads were incubated with samples and with TGF‐β, IL‐1 β or TNF‐α standard curves, and then with biotin‐conjugated secondary antibodies and streptavidin‐phycoerythrin. Fluorescence was read with a cytofluorimeter (CyFlow^®^ Space; Partec, Carate Brianza, MB, Italy). Values are indicated as mean ± S.E.M. of six individual mice from each group and expressed as pg/μg of total proteins determined over an albumin standard curve.

### Western blot determination of iNOS, COX‐2 and pSMAD3 level expression

Tissue samples (30 μg protein per lane) were subjected to 8% SDS‐PAGE, transferred to nitrocellulose membranes and incubated overnight (4°C) with polyclonal antibody anti‐iNOS1 raised in rabbit, diluted 1:1000 (Merck‐Millipore, Darmstadt, Germany) in PBS‐T (20 mM Tris‐HCl buffer, 150 mM NaCl and 0.05% Tween 20), and with anti‐COX‐2 rabbit polyclonal antibody (Cayman Chemical Co., Ann Arbor, MI, USA) diluted 1:150 in PBS‐T. After several rinses with PBS‐T, membranes were incubated with anti‐rabbit IgG conjugated with horseradish peroxidase, diluted 1:5000 in PBS‐T at RT for 1 hr. The bands were visualized by enhanced chemiluminescence (ECL) and quantified by densitometric analysis. For SMAD3 signalling pathway analysis, 150 μg of total proteins was loaded onto 10% SDS‐PAGE gel, transferred to nitrocellulose membranes, incubated overnight (4°C) with pSMAD3 and SMAD3 [1:1000 in 5% bovine serum albumin (BSA), TBS‐T; Cell Signaling Technology, Danvers, MA, USA] and successively, incubated with anti‐rabbit secondary antibody (1:2000 in 5% BSA in TBS‐T).

### Determination of 8‐Hydroxy‐2′‐deoxyguanosine

Frozen lung samples were thawed at room temperature, and cell DNA isolation was performed as previously described 42 with minor modifications. Briefly, lung samples were homogenized in 1 ml of 10 mM PBS, pH 7.4, sonicated on ice for 1 min., added with 1 ml of 10 mmol/l Tris‐HCl buffer, pH 8, containing 10 mmol/l EDTA, 10 mmol/l NaCl, and 0.5% SDS, incubated for 1 hr at 37°C with 20 μg/ml RNase 1 (Sigma‐Aldrich, Saint Louis, MO, USA) and overnight at 37°C under argon in the presence of 100 μg/ml proteinase K (Sigma‐Aldrich). The mixture was extracted with chloroform/isoamyl alcohol (10/2 v/v). DNA was precipitated from the aqueous phase with 0.2 volumes of 10 mmol/l ammonium acetate, solubilized in 200 μl of 20 mmol/l acetate buffer, pH 5.3, and denatured at 90°C for 3 min. The extract was then supplemented with 10 IU of P1 nuclease (Sigma‐Aldrich) in 10 μl and incubated for 1 hr at 37°C with 5 IU of alkaline phosphatase (Sigma‐Aldrich) in 0.4 mol/l phosphate buffer, pH 8.8. All of the procedures were performed in the dark under argon. The mixture was filtered by an Amicon Micropure‐EZ filter (Merck‐Millipore), and 50 μl of each sample was used for 8‐hydroxy‐2‐deoxyguanosine (8‐OH*d*G) determination using a ELISA kit (JalCA, Shizuoka, Japan), following the instructions provided by the manufacturer. The absorbance of the chromogenic product was measured at 450 nm and expressed as ng/mg of DNA. The results were calculated from a standard curve based on a 8‐OH*d*G solution. The values are expressed as ng 8‐OH*d*G/ng total DNA.

### Statistical analysis

For each assay, data were reported as mean values (± S.E.M.) of individual average measures of the different animals per group. Significance of differences among the groups was assessed by one‐way anova followed by Newman–Keuls post hoc test for multiple comparisons. Calculations were made with Prism 5 statistical software (GraphPad Software, Inc., San Diego, CA, USA). A probability value (*P*) of <0.05 was considered significant.

## Results

### Bleomycin and PARP activity in the lungs: effects of HYDAMTIQ

**Figure 1:**
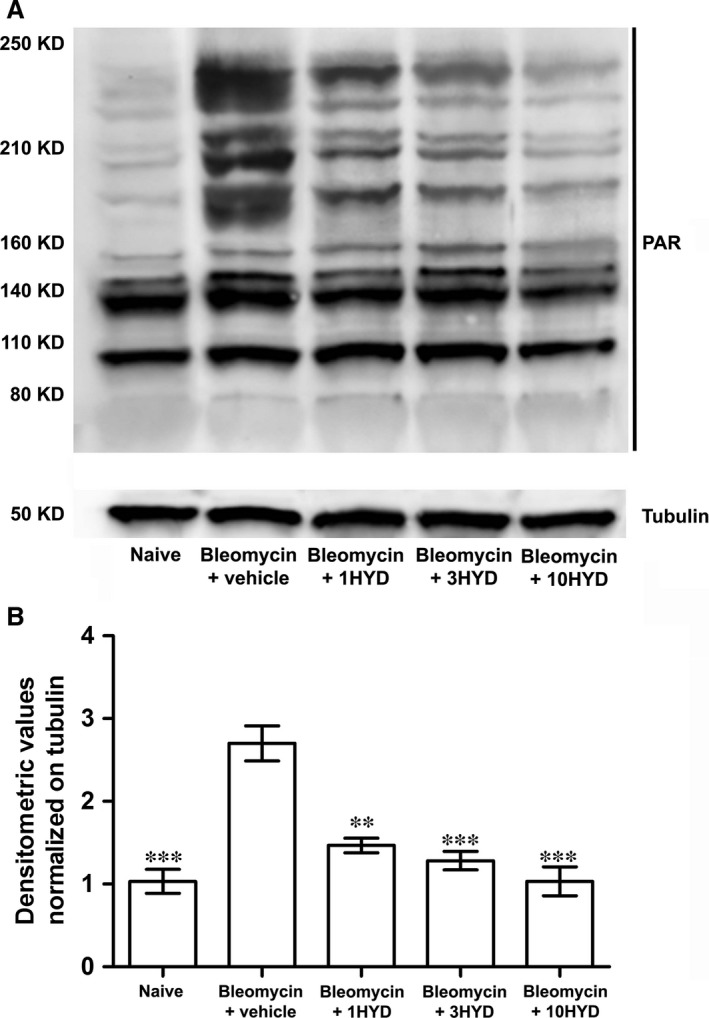
PARP activity. (**A**) Western blot analysis of PARylated protein content in lung samples from each experimental group. (**B**) Densitometric analysis was normalized with tubulin (*n* = 10 animals per group). **P < 0.01 and ***P < 0.001 *versus* bleomycin + vehicle. 1HYD, 3HYD, 10HYD: 1, 3, 10 mg/kg of HYDAMTIQ.

It has been previously reported that bleomycin may activate PARP activity in mouse lung *in vitro* 43. In the first series of experiments, we evaluated the effects of intratracheal administration of bleomycin (0.05 IU), a dose able to lead to pulmonary fibrosis 38 on PARP activity, and Figure 1 shows that PARylated protein levels, the products of PARP activity, were significantly increased in lung homogenates of mice treated with bleomycin. The treatment of the mice with bleomycin plus HYDAMTIQ (1, 3 and 10 mg/kg/day) significantly prevented this effect.

### Bleomycin and lung morphology and function

**Figure 2:**
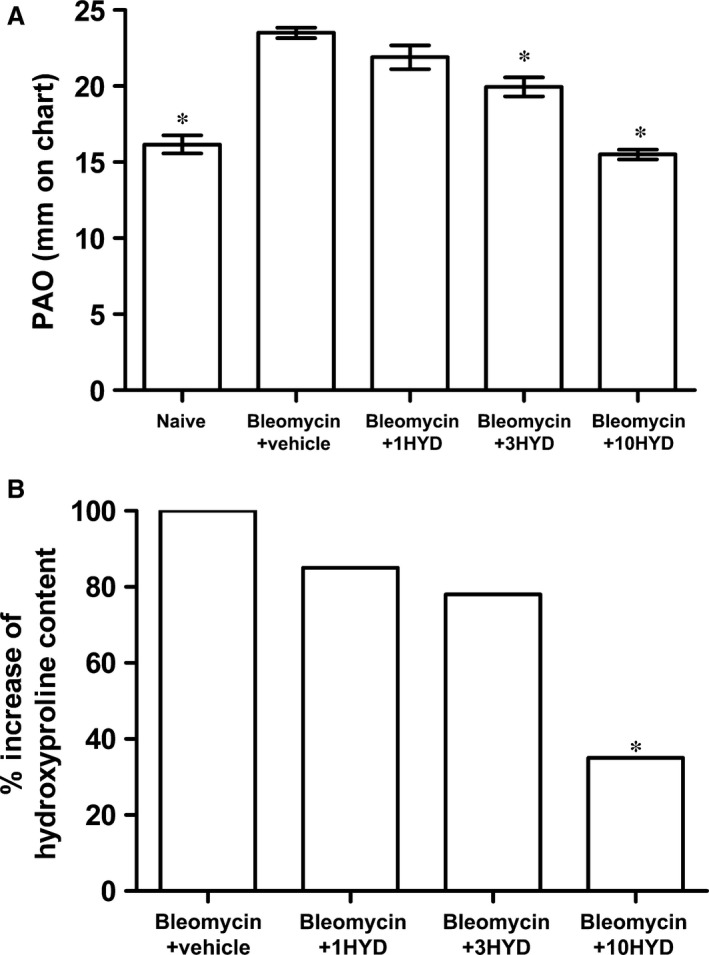
Lung function and hydroxyproline content. (**A**) Lung resistance to airflow measured through the evaluation of pressure at airway opening (PAO) (*n* = 10 animals per group). (**B**) Percentage increase of hydroxyproline content. Basal content of hydroxyproline in controls (naïve): 15.0 ± 1 μg/ml; bleomycin + vehicle: 26.85 ± 1.05 μg/ml. Data are mean ± S.E.M. *P < 0.05 *versus* bleomycin + vehicle. Bleo: bleomycin; 1HYD, 3HYD, 10HYD: 1, 3, 10 mg/kg of HYDAMTIQ.

Intratracheal bleomycin causes a significant increase in lung weight and airway stiffness leading to a clear‐cut elevation of the PAO 35. In the present series of experiments, we evaluated PAO in controls, bleomycin‐treated mice and bleomycin plus HYDAMTIQ‐treated mice. Figure 2A shows that bleomycin treatment increased PAO from 16.8 ± 0.50 to 23.4 ± 0.38 mm on chart (*P* < 0.01), while the administration of HYDAMTIQ, 1, 3 and 10 mg/kg/day for 21 days, dose dependently attenuated the effects of bleomycin on PAO. Moreover, lung static compliance was evaluated, and it was significantly higher in bleomycin‐treated mice (0.095 ± 0.005 ml/cm H_2_O, *n* = 6) compared with controls (0.052 ± 0.003 ml/cm H_2_O, *n* = 6). HYDAMTIQ treatment at the highest dose of 10 mg/kg reduced significantly the increase in static compliance (0.073 ± 0.002, *n* = 6).

Figure 2B shows the percentage increase in hydroxyproline content in lung tissue homogenates. In controls (Naïve), the basal content of hydroxyproline was 15.0 ± 1 μg/ml, while in vehicle 26.85 ± 1.05 μg/ml. The treatment with HYDAMTIQ prevented this effect in a dose‐dependent manner, strengthening our morphological findings on lung fibrosis.

### Lung histology

**Figure 3:**
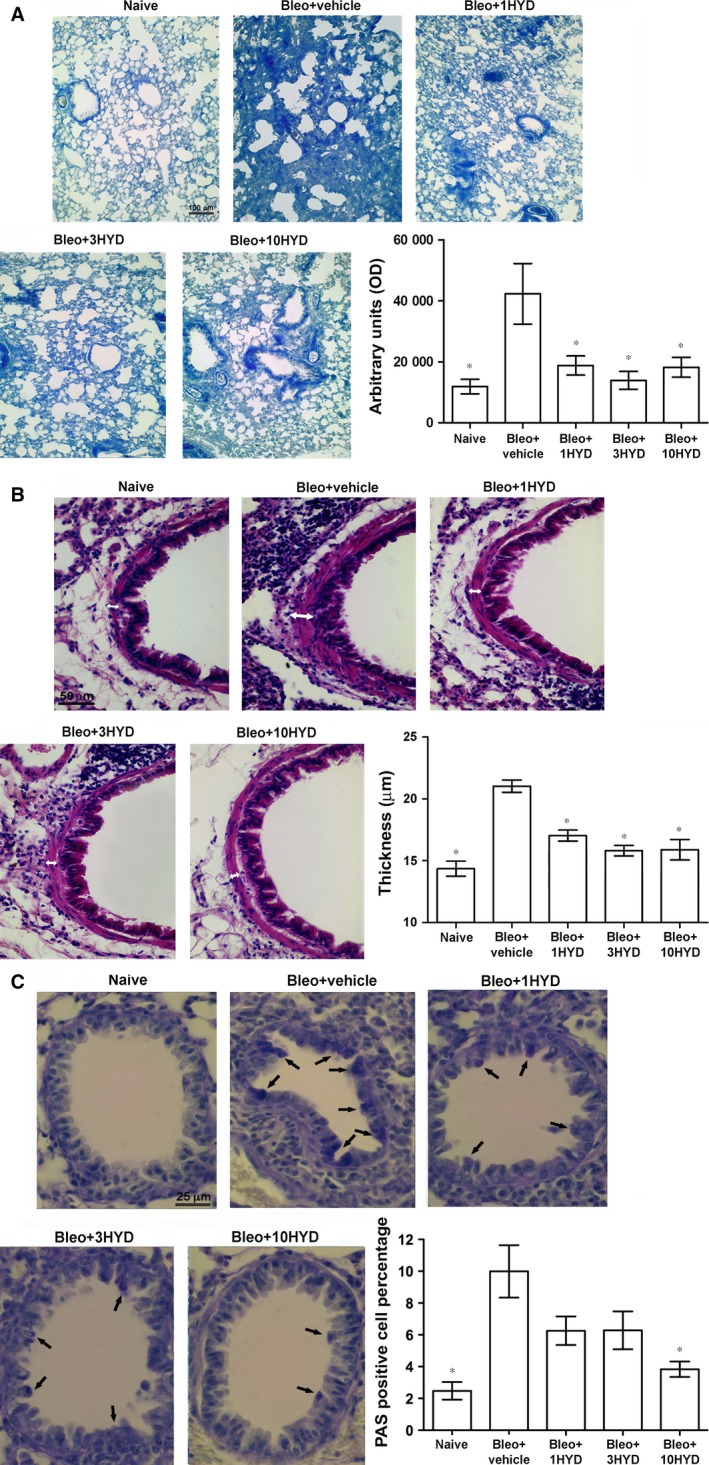
Lung histology. (**A**) Histopathological evaluation of fibrosis by Azan‐stained lung analysis. By computer‐aided densitometry analysis, it is possible to obtain a semi‐quantitative measure of this accumulation. *n* = 10 animals per group. Data are mean ± S.E.M. *P < 0.05 *versus* bleomycin + vehicle. 1HYD, 3HYD, 10HYD: 1, 3, 10 mg/kg of HYDAMTIQ. (**B**) Histopathological evaluation of airway remodelling by haematoxylin and eosin staining. *n* = 10 animals per group. Data are mean ± S.E.M. *P < 0.05 *versus* bleomycin + vehicle. 1HYD, 3HYD, 10HYD: 1, 3, 10 mg/kg of HYDAMTIQ. (**C**) Goblet cell number in PAS‐stained lung sections (see the arrows) is evaluated in each experimental group. *n* = 10 animals per group. Data are mean ± S.E.M. *P < 0.05 *versus* bleomycin + vehicle. 1HYD, 3HYD, 10HYD: 1, 3, 10 mg/kg of HYDAMTIQ.

The results reported in Figure 3A clearly show that intratracheal administration of bleomycin increased collagen accumulation in interstitial lung spaces with almost complete destruction of the alveolar architecture. HYDAMTIQ treatment reduced these pathological changes and the lesions were drastically reduced.

In order to obtain information on the status of the bronchial smooth muscle layer, we measured its thickness using a morphometrical analysis in haematoxylin and eosin–stained preparations. To evaluate bronchial mucosa goblet cell hyperplasia, PAS‐stained preparations were utilized. As expected, the thickness of the airway smooth muscle layer was increased in mice treated with bleomycin. HYDAMTIQ treatment reduced this damage (Fig. 3B). Similarly, the percentage of PAS‐positive goblet cells over total bronchial epithelial cells significantly increased in mice treated with bleomycin (Fig. 3C). In HYDAMTIQ‐treated animals, this marker of mucosal damage was significantly reduced.

### TGF‐β signalling pathway

**Figure 4:**
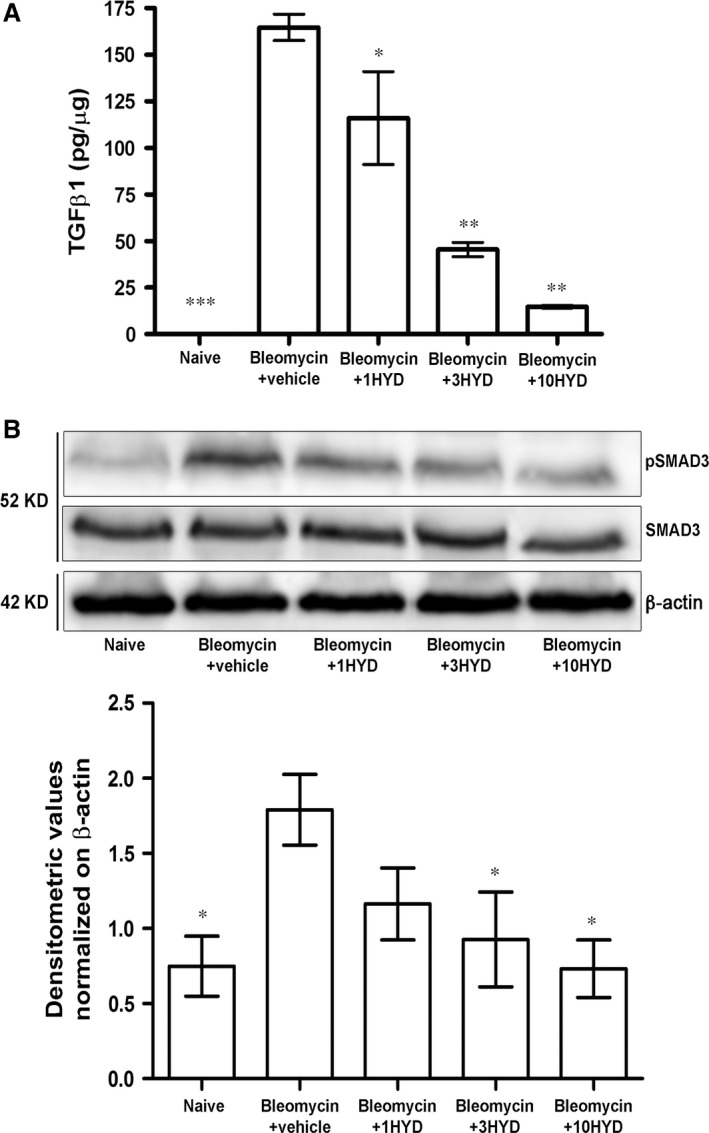
Evaluation of TGF‐β signalling pathway. (**A**) Determination of the pro‐fibrotic marker TGF‐β. Values are expressed as pg of protein/μg of total proteins (*n* = 10 animals per group). Data are mean ± S.E.M. *P < 0.05, **P < 0.01 and ***P < 0.001 *versus* bleomycin + vehicle. (**B**) pSMAD3 expression level, assayed by Western blotting. Densitometric analysis was normalized on β‐actin (*n* = 10 animals per group). Data are mean ± S.E.M. *P < 0.05 *versus* bleomycin + vehicle. 1HYD, 3HYD, 10HYD: 1, 3, 10 mg/kg of HYDAMTIQ.

It has been repeatedly proposed that an increased TGF‐β expression and an increased TGF‐β signalling through SMAD pathways contribute to the establishment and development of pulmonary fibrosis 44, 45, 46. In this study, we confirmed that bleomycin caused a large increase of TGF‐β lung levels (from 0.04 ± 0.002 to 165 ± 7.1 pg/ml, *P* < 0.001). HYDAMTIQ (1, 3 and 10 mg/kg/day) treatment reduced this increase (Fig. 4A). At the highest dose (10 mg/kg), TGF‐β was not significantly different in comparison to the control level. Phosphorylation of SMAD3 is an expected consequence of TGF‐β increase, and we evaluated SMAD3 and pSMAD3 proteins in lung homogenates with Western blot analysis. Bleomycin‐treated animals presented a large increase of pSMAD3 in the lungs, while in bleomycin plus HYDAMTIQ‐treated animals, the levels of the protein returned towards control values (Fig. 4B).

### Fibroblast activation: αSMA

**Figure 5:**
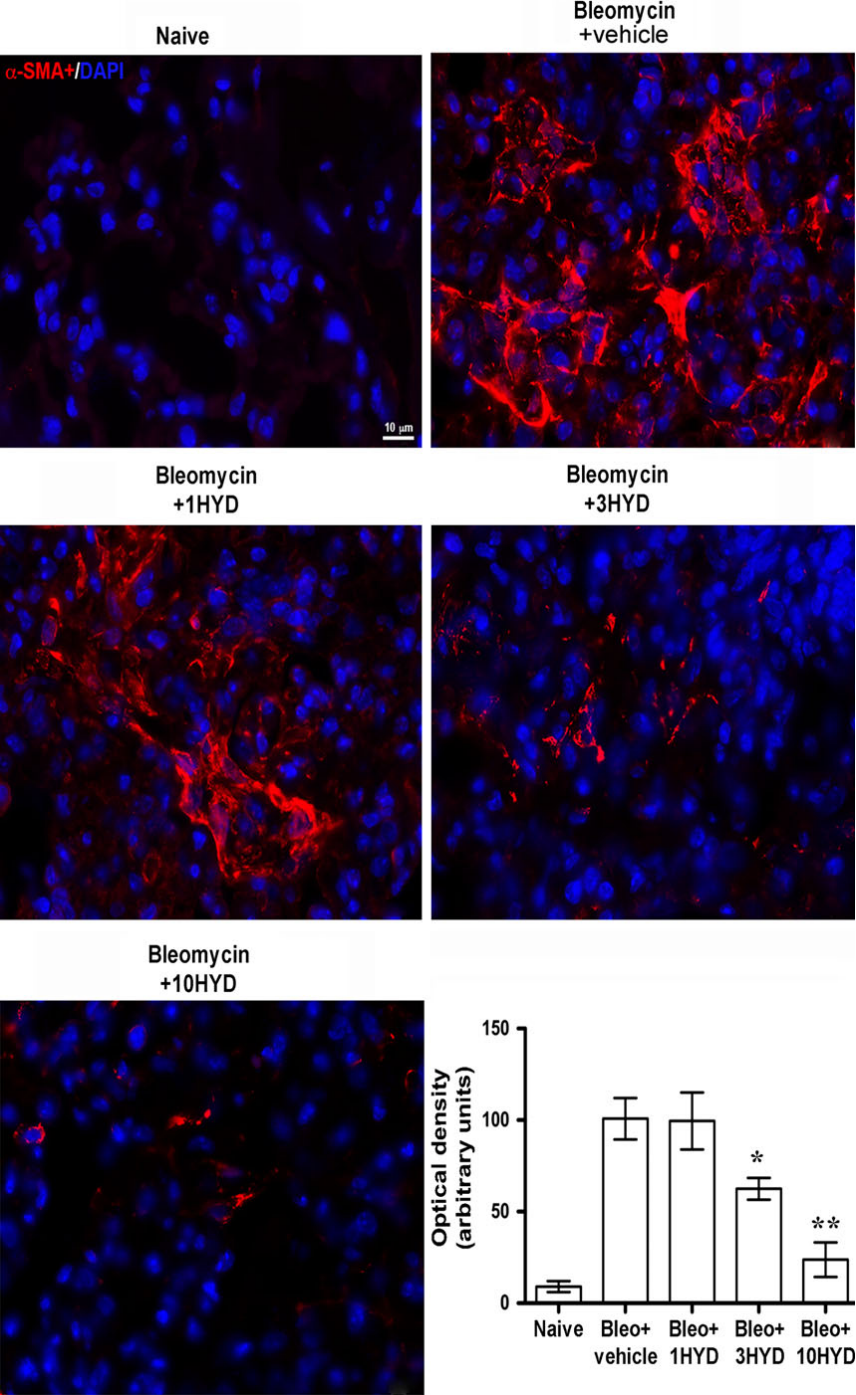
Evaluation of fibroblast activation. Immunofluorescence staining of lung tissue sections labelled with alpha‐smooth muscle actin (αSMA) (red) and nuclei (blue) counterstained with DAPI (40×). Images in the panels show the inhibition of αSMA expression, a marker of the transformation of fibroblasts into myofibroblasts (*n* = 10 animals/group). *P < 0.05, **P < 0.001 *versus* bleomycin + vehicle. Bleo: bleomycin; 1HYD, 3HYD, 10HYD: 1, 3, 10 mg/kg of HYDAMTIQ.

Transforming growth factor‐β signalling has been reported to regulate the expression of αSMA, a marker of fibroblast activation and myofibroblast differentiation 47, 48. We used immunofluorescence analysis to evaluate the expression of αSMA in lungs of bleomycin‐exposed mice. A large increase of αSMA levels was detected (Fig. 5) in bleomycin‐exposed animals. In the lungs of bleomycin plus HYDAMTIQ‐treated animals, the levels of αSMA were drastically reduced. This result clearly indicates that the PARP inhibitor reduces fibroblast activation and myofibroblast differentiation in bleomycin‐exposed mice (Fig. 5).

### Determination of pro‐inflammatory markers

**Figure 6:**
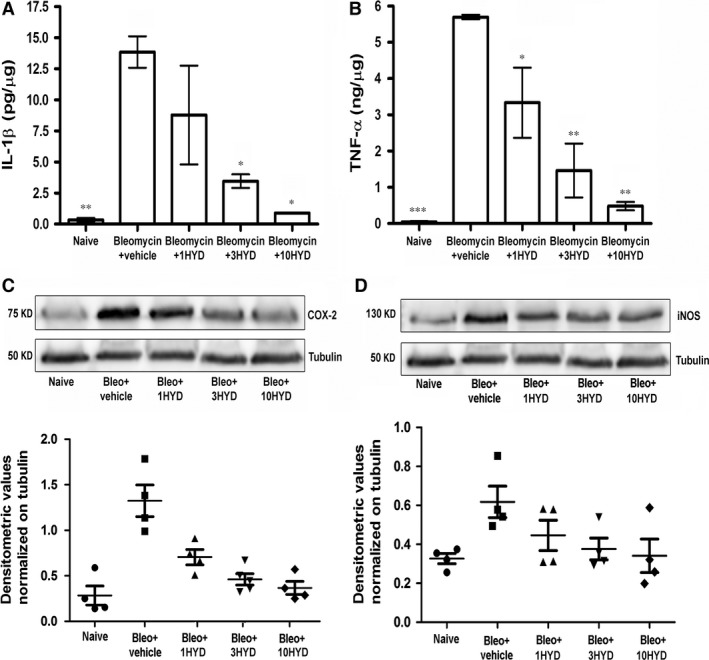
Determination of pro‐inflammatory markers. (**A** and **B**) Analysis of IL‐1β and TNF‐α content (respectively) in the supernatant of lung tissue homogenates. The values are expressed as pg or ng/μg of total proteins (*n* = 10 animals per group). Values are mean ± S.E.M. *P < 0.05 and **P < 0.01 *versus* bleomycin + vehicle. (**C** and **D**) Levels of pro‐inflammatory proteins iNOS and COX‐2 (respectively) are evaluated by Western blot analysis from total cell extracts of lung samples homogenates. The densitometric analysis was normalized to tubulin and each point report data obtained in a single animal (*n* = 4 animals per group). Bleo: bleomycin; 1HYD, 3HYD, 10HYD: 1, 3, 10 mg/kg of HYDAMTIQ.

Chronic inflammation may drive fibrotic progression in animal model of fibrosis, although the contribution of inflammation to fibrosis in the clinical manifestation of the disease has been repeatedly questioned. We evaluated the late (21 days after challenge) inflammatory response to bleomycin by measuring the cytokines IL‐1β and TNF‐α (Fig. 6A and B) and the pro‐inflammatory enzymes iNOS and COX‐2 in lung homogenates (Fig. 6C and D). Bleomycin treatment increased IL‐1β and TNF‐α production in the lungs (from: 0.35 ± 0.15 to 13.8 ± 1.3 pg/μg protein and from 0.045 ± 0.02 to 5.7 ± 0.06 ng/μg protein, respectively). HYDAMTIQ treatment reduced these increases in a dose‐dependent manner. In a similar manner, bleomycin increased iNOS and COX‐2 protein expression which was dampened by HYDAMTIQ treatment (Fig. 6C and D). Overall, these results suggest that HYDAMTIQ not only attenuated bleomycin‐induced lung fibrosis, but also the bleomycin‐induced inflammatory responses.

### Evaluation of oxidative stress parameter

**Figure 7:**
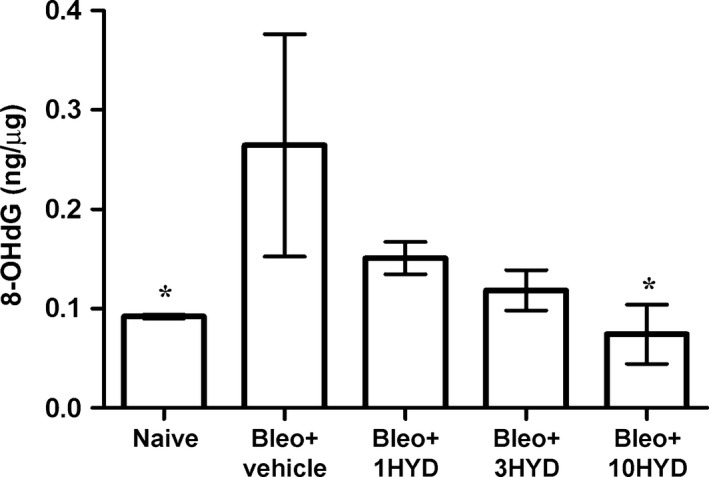
Evaluation of oxidative stress parameter in lung tissue. Levels of 8‐OH *d*G, a marker of free radicals‐induced DNA damage (*n* = 10 animals per group). Values are mean ± S.E.M. *P < 0.05 *versus* bleomycin + vehicle. Bleo: bleomycin; 1HYD, 3HYD, 10HYD: 1, 3, 10 mg/kg of HYDAMTIQ.

Measurements of 8‐OH*d*G (Fig. 7), a marker of oxidative DNA damage, showed that bleomycin treatment increased significantly 8‐OH*d*G level (from 0.092 ± 0.002 to 0.264 ± 0.112 ng/μg DNA, *P* < 0.05), while HYDAMTIQ treatment caused a dose‐dependent reduction of this parameter, suggesting that PARP activity contributes to the expression of the oxidative lung damage.

## Discussion

We report here that PARP inhibition reduces the functional and structural features of bleomycin‐induced lung pathology in mice. Since intratracheal bleomycin administration is a well‐characterized and widely accepted model for lung fibrosis 36, 49, 50, we suggest that agents able to inhibit PARP could represent a new valuable therapeutic strategy for the treatment of this devastating disorder. The bleomycin‐induced lung fibrosis is caused by an acute damage, while IPF is due to a persistent imbalance of reparative and immunologic processes in genetically predisposed patients, and, in general, it is not always correct to extrapolate the results obtained in animal models to clinical situations.

The current therapeutic approach to IPF is oriented towards drugs targeting the pathways of fibroblasts activation, myofibroblast differentiation with increased αSMA expression and extracellular matrix accumulation 51. The cytokine TGF‐β has been proposed to play key roles in this process 52, 53, and drugs able to modulate TGF‐β expression and/or signalling seem to be active in reducing fibroblast activation, αSMA synthesis 54 and the clinical progression of the disease 55, 56. The involvement of TGF‐β in pulmonary fibrosis has been well documented with elevated growth factor levels both in animal models 57 and in patients with IPF 14. It has also been demonstrated that TGF‐β overexpression induces lung fibrosis 13 while the administration of TGF‐β neutralizing antibodies or of inhibitors of TGF‐β signalling pathway prevents lung fibrosis 55, 56. Fibroblasts contribute to the onset of the disease by directly secreting TGF‐β, thus inducing differentiation of myofibroblasts 58, profoundly affecting epithelial and airway smooth muscle cells 59 and increasing extracellular matrix secretion. Recently, it has been shown that PARP‐1 activity is required to allow lung fibroblasts activation and proliferation with increased expression of αSMA, which seems to play a major role in lung fibrosis 16. Our findings show that lung PARP activity and TGF‐β levels significantly increased after bleomycin (Figs 1 and 4), and PARP inhibition drastically reduced both PARP activity and TGF‐β levels in a dose‐dependent manner (Fig. 4A). In line with these observations, mice treated with bleomycin plus a PARP inhibitor have a reduced TGF‐β signalling pathway activation, as shown by measuring lung levels of SMAD 3 and of pSMAD 3 (Fig. 4). These findings are in line with literature data, suggesting that an elevated PARP activity is necessary to increase αSMA expression after stimulation of the TGF‐β/SMAD pathway (Figs 3 and 5) 15, 16. Thus, PARP inhibitors, by reducing TGF‐β expression and dampening the TGF‐β/SMAD transduction pathway, are able to prevent fibroblast activation, αSMA and extracellular matrix protein deposition and finally, fibrosis development.

The model of pulmonary fibrosis we used is well characterized and includes a first phase (7–9 days) of acute inflammation, followed in the next 2 weeks by a chronic inflammatory infiltrate and finally pulmonary fibrosis 50. While anti‐inflammatory drugs are poorly effective in ameliorating the clinical course of the disease 60 and are not able to prevent and/or delay the onset of insufficient breathing in experimental models 36, we cannot rule out that the cytoprotective and anti‐inflammatory actions of PARP inhibitors may contribute to the results we obtained. In our experimental setting, the treatment with HYDAMTIQ during the onset of bleomycin‐induced inflammatory process may prevent the pro‐fibrotic progression. However, further studies are necessary to evaluate if HYDAMTIQ maintains its beneficial effects when the treatment is started when the inflammatory and pro‐fibrotic process is already established. In this study, we found that several inflammatory markers (including IL‐1β and TNF‐α levels with iNOS and COX 2 expression) and 8‐OH*d*G, an index of oxidative stress and DNA damage, were significantly reduced in lungs of bleomycin plus PARP inhibitor‐treated mice in a dose‐dependent manner (Figs 6 and 7). The importance of these events in the overall beneficial actions of PARP inhibition is not easy to quantify. A specific anti‐fibrotic effect of PARP inhibitors has been shown also in models of fibrotic disorders affecting heart 23, 24, liver 25, peritoneum 61 and vessels 15, 26, suggesting that PARP is involved in fibrotic processes beyond its involvement in oxidative damage and inflammation. In fact, PARP activity in lung fibroblasts isolated from IPF patients was significantly higher than that in cells isolated from control subjects 16.

In order to inhibit PARP activity, we administered HYDAMTIQ, a potent and specific inhibitor previously characterized in our laboratory 31, 34. The inhibitor was used at doses that are effective in reducing brain infarct sizes and granulocyte infiltration in stroke models 31 and in ameliorating functional, morphological and biochemical lung abnormalities induced by repeated antigen exposure in allergen‐induced asthma like reaction in guinea pigs 33. These observations suggest that HYDAMTIQ, similarly to other PARP inhibitors, reduces oxidative‐stress‐induced tissue damage and attenuates the expression of pro‐inflammatory mediators 62, 63, 64, 65, 66, 67. The relative importance of each of these multiple events in the overall beneficial actions of HYDAMTIQ in pulmonary fibrosis is not easy to quantify.

Idiopathic pulmonary fibrosis treatment is now based on the use of either pirfenidone, an agent that reduces TGF‐β signalling pathway and it has also anti‐inflammatory actions 30, or nintedanib, a tyrosine kinase inhibitor able to reduce the transduction pathway of a number of growth factor receptors, and to reduce the transduction pathways leading to cell activation and proliferation 4. Both agents have recently been introduced into clinical practice on the basis of clinical trials showing their ability to reduce the rate of progression of the disease evaluated by testing the forced vital capacity 9. The therapeutic improvement is modest, and it is still not clear whether these agents have a clinically meaningful efficacy in long‐term patient survival 9. Hence, there remains a considerable need for continued research in drug discovery in this devastating disorder 4, and a large number of compounds and pathways are currently investigated to improve the fate of IPF patients. Since PARP inhibitors are clinically used in cancer therapy and are well tolerated, their use in IPF patients with the aim of reducing disease progression and improving patient survival seems a rational proposal 68. It should be mentioned that PARP is activated by oxidative stress‐induced DNA damage and triggers cell death 17, 67, 69 and inflammation pathways 70, 71, 72. Both cell death and inflammation possibly activate the first phases of lung fibrosis. PARP inhibitors could, therefore, be therapeutically useful because their action is not limited to the TGF‐β/SMAD pathway but is extended to cellular protection and reduction of inflammatory mediator expression 73, which contributes to the damage of most cell types present in the airways and lung parenchyma.

## Conflict of interest

Flavio Moroni has patent applications on HYDAMTIQ and other PARP inhibitors.

## Author contribution

LL, FM, EM and GM designed the research study. LL, CL, AP, MD, GB and LC performed the research. AP and LL analysed the data. LL, FM, EM and GM wrote the paper.
